# Supplementary figures and images for: Benthic foraminifera as bio-indicators of natural and anthropogenic conditions in Roscoff Aber Bay (Brittany, France)
Source: PLoS One. 2024 Oct 31;19(10):e0309463. doi: 10.1371/journal.pone.0309463 (PMC11527215; doi:10.1371/journal.pone.0309463)

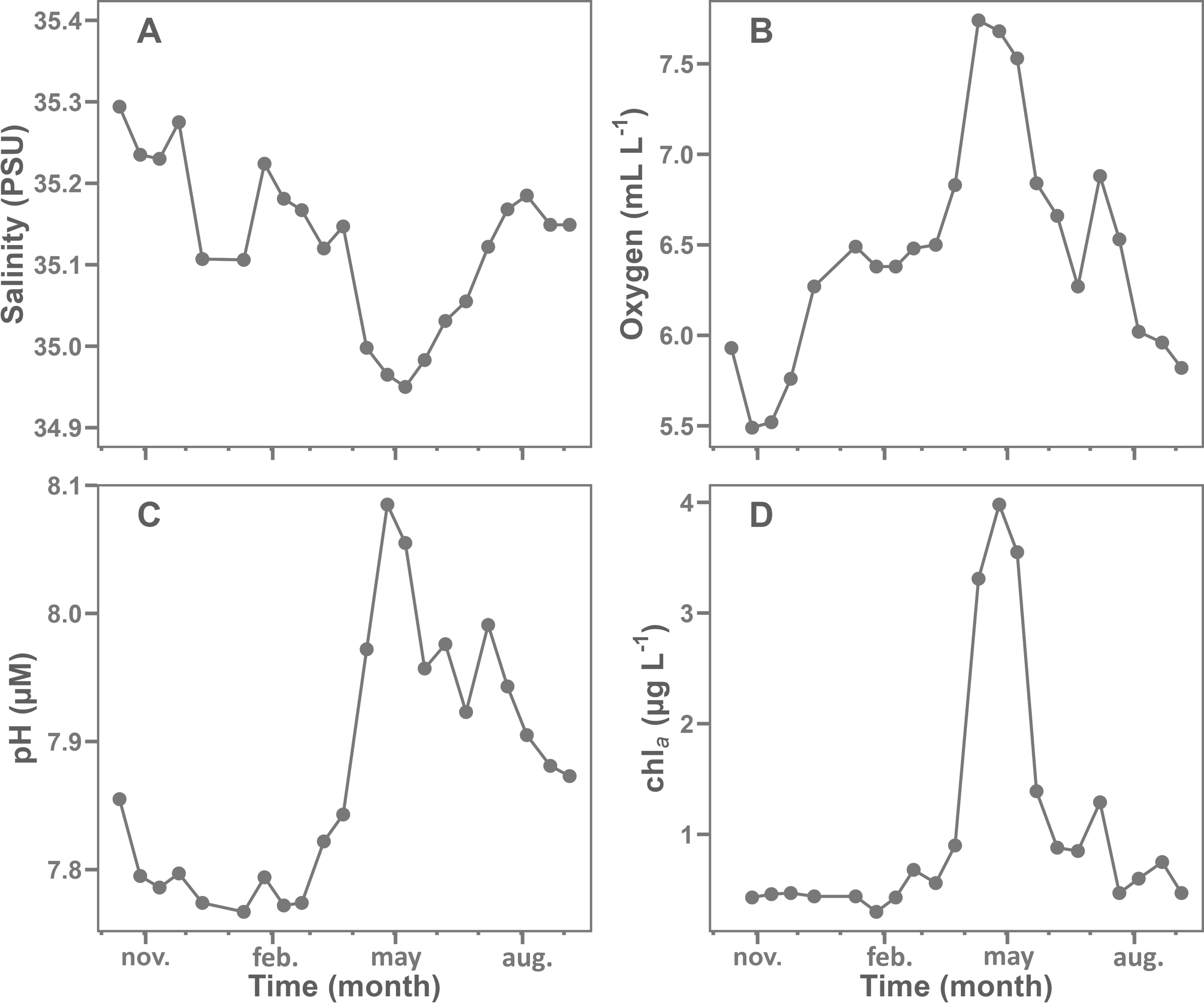

Supplement: S1 Fig — The data were extracted from the SOMLIT database (Service d’Observation en Milieu Littoral; www.somlit.fr) on 5 October 2022. (TIF) [file pone.0309463.s001.tif]

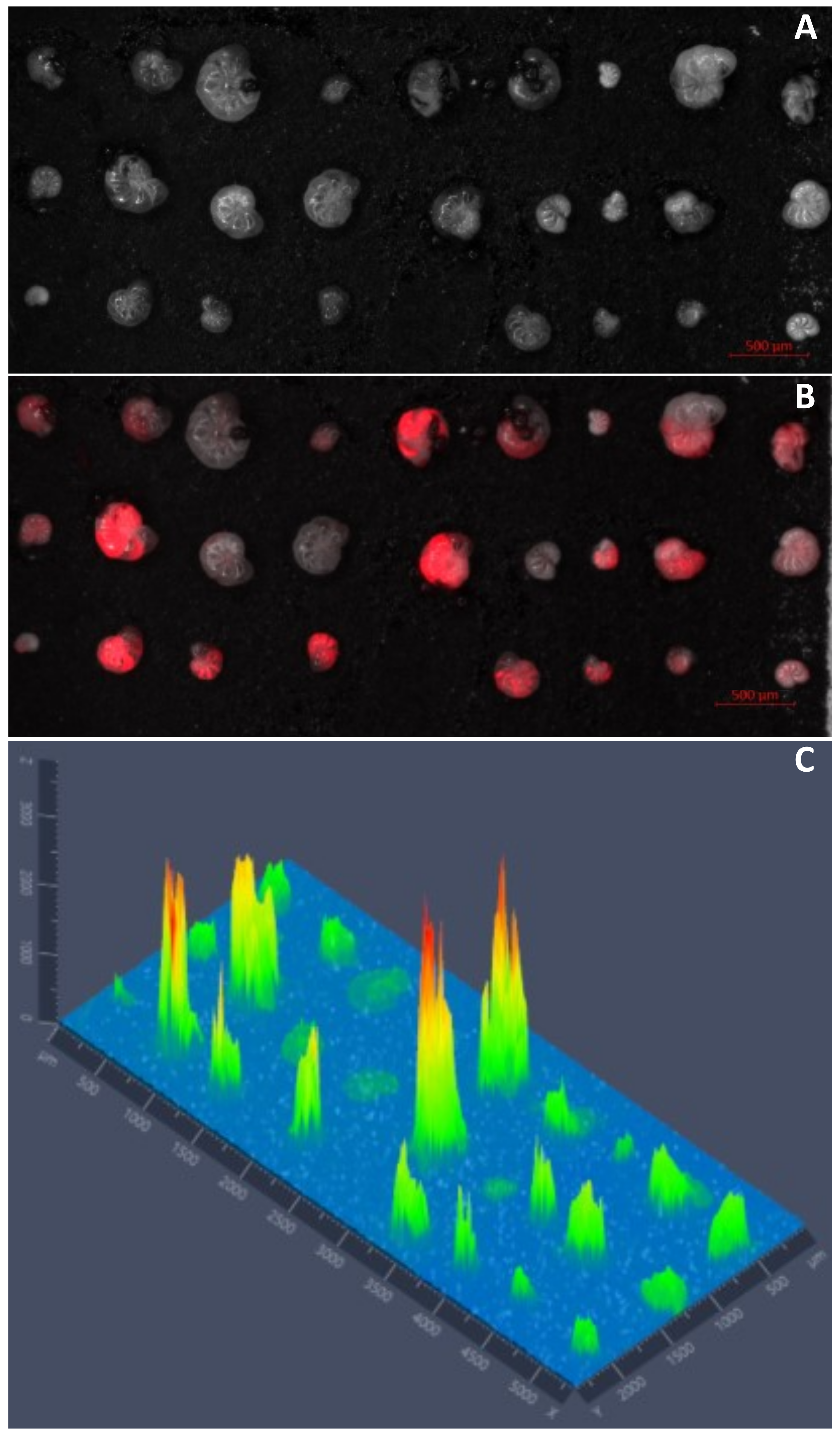

Supplement: S2 Fig — (A) and red fluorescence channels (Ex 559–585 nm, Em 600–690 nm, BeamSplitter 590 nm) (B) and an illustration of a density plot for red fluorescence (C). (TIF) [file pone.0309463.s002.tif]

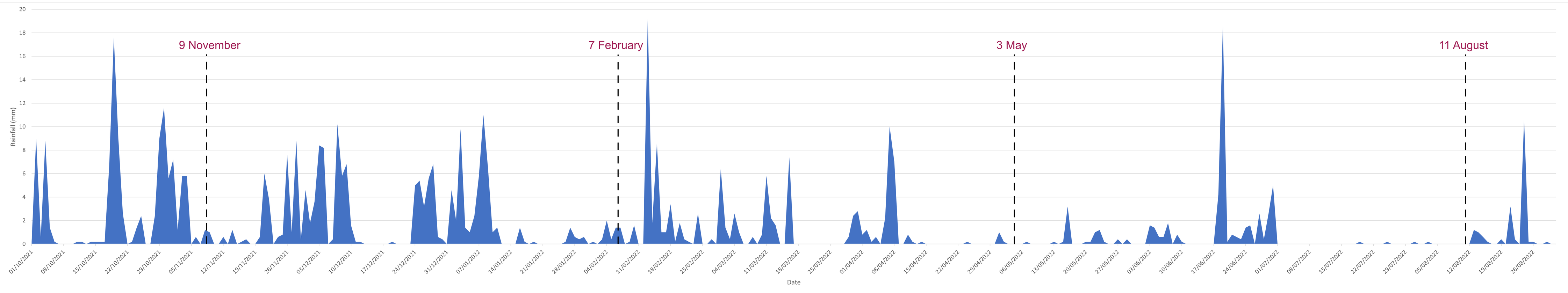

Supplement: S3 Fig — The period October 2021 to August 2022 from the infoclimat.fr/climatologie/globale/31-aout/morlaix/000AW.html website. (TIF) [file pone.0309463.s003.tif]

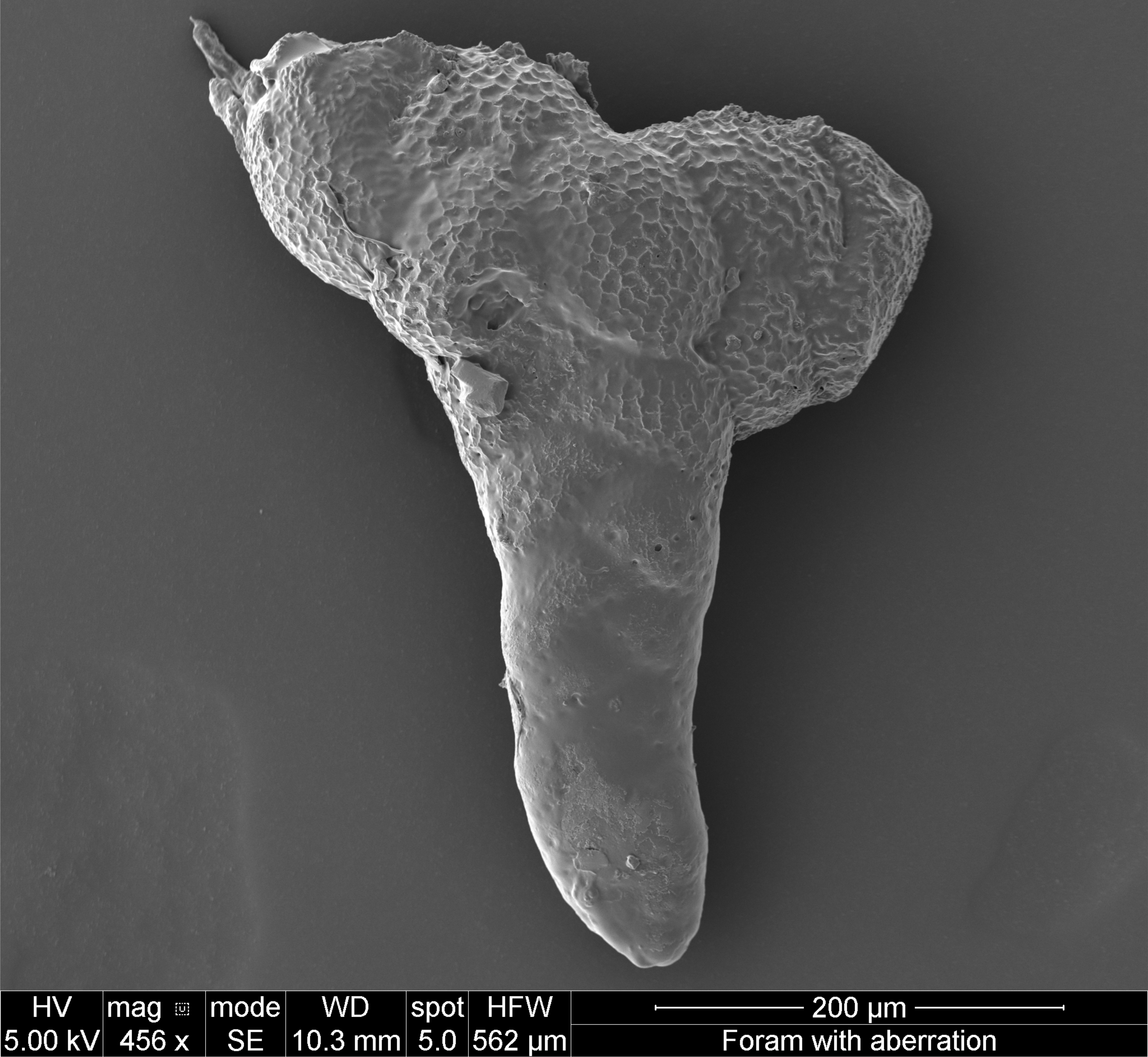

Supplement: S4 Fig — (TIF) [file pone.0309463.s004.tif]
