# Supplementary material for: Benthic foraminifera as bio-indicators of natural and anthropogenic conditions in Roscoff Aber Bay (Brittany, France)
Source: PLoS One. 2024 Oct 31;19(10):e0309463. doi: 10.1371/journal.pone.0309463 (PMC11527215; doi:10.1371/journal.pone.0309463)
Supplement: Attachment — Submitted filename: PONE-D-24-06208_reviewer.docx [file pone.0309463.s005.docx]

PLOS ONE

Benthic Foraminifera as bio-indicators of natural and anthropogenic stresses

--Manuscript Draft--

| Manuscript Number: | PONE-D-24-06208 |
| --- | --- |
| Article Type: | Research Article |
| Full Title: | Benthic Foraminifera as bio-indicators of natural and anthropogenic stresses |
| Short Title: | Bio-indicator foraminiferal species |
| Corresponding Author: | Edwin Dache  Ifremer Centre de Brest: Ifremer Centre de Bretagne Plouzané, FRANCE |
| Keywords: | benthic foraminifera, bioindicators, seasonal effect, anthropic impact, pollution monitoring |
| Abstract: | Benthic foraminifera are good environmental bio-indicators of both natural and anthropogenic stresses in marine environments. In our study, we investigated living benthic foraminifera from a coastal environment in the Roscoff Aber Bay (Brittany, France) at eight sampling sites subjected to natural variations (freshwater inputs, tides) and/or anthropogenic impacts (pollution). These sites were studied over four seasons in 2021-2022 (November, February, May, August). The main objective of this work is to analyse the spatial distribution of foraminiferal populations within and between sampling sites during different seasons and identify opportunistic species and those tolerant to anthropogenic impacts. To this end, sedimentological and biogeochemical characteristics of the sediments were examined by measuring grain size, temperature, oxygen, salinity, pH, pigment concentration (chlorophyll a and phaeopigments), total organic carbon (TOC), as well as the isotopic ratio of carbon (δ13C), nitrogen (δ15N) and sulfide (δ34S). Using these environmental data as potential driving factors, four domains were distinguished among the sampling sites: open water, terrestrial, oligotrophic, and eutrophic domains, showing an increasing gradient of organic supply as well as very contrasted microbial activities highlighted by carbon and sulphide isotopic ratios. Our faunal results show a lower abundance but highest diversity of foraminiferans in the harbour associated with the dominance of Haynesina germanica suggesting its particular tolerance to anthropogenic impacts. In the open water domain, Ammonia beccarii and Elphidium crispum were the most abundant species while Quinqueloculina seminula was the dominant species in the terrestrial domain. These preliminary data show an organic enrichment in the harbour due to anthropogenic activities that do not seem to significantly affect faunal diversity. The use of benthic foraminiferal species in the Roscoff bay appears to be a good proxy to evaluate marine environment health under various natural/anthropogenic stresses. |
| Order of Authors: | Edwin Dache |
|  | Pierre-Antoine Dessandier |
|  | Ranju Radhakrishnan |
|  | Valentin Foulon |
|  | Loic Michel |
|  | Colomban de Vargas |
|  | Jozee Sarrazin |
|  | Daniela Zeppilli |
| Additional Information: |  |
| Question | Response |
| Financial Disclosure  Enter a financial disclosure statement that | The author(s) received no specific funding for this work. |

| describes the sources of funding for the work included in this submission. Review the [submission guidelines](http://journals.plos.org/plosone/s/submission-guidelines#loc-financial-disclosure-statement) for detailed requirements. View published research articles from [PLOS ONE](https://journals.plos.org/plosone/) for specific examples.  This statement is required for submission and will appear in the published article if the submission is accepted. Please make sure it is accurate. |  |
| --- | --- |
| Funded studies Enter a statement with the following details: - Initials of the authors who received each award - Grant numbers awarded to each author - The full name of each funder - URL of each funder website - Did the sponsors or funders play any role in the study design, data collection and analysis, decision to publish, or preparation of the manuscript? |  |
| Did you receive funding for this work? |  |
| Competing Interests  Use the instructions below to enter a competing interest statement for this submission. On behalf of all authors, disclose any [competing interests](http://journals.plos.org/plosone/s/competing-interests) that could be perceived to bias this work—acknowledging all financial support and any other relevant financial or nonfinancial competing interests.  This statement is required for submission and will appear in the published article if the submission is accepted. Please make sure it is accurate and that any funding sources listed in your Funding Information later in the submission form are also declared in your Financial Disclosure statement.  View published research articles from [PLOS ONE](http://journals.plos.org/plosone/) for specific examples. | No competing interests |

| NO authors have competing interests Enter: The authors have declared that no competing interests exist. Authors with competing interests Enter competing interest details beginning with this statement: I have read the journal's policy and the authors of this manuscript have the following competing interests: [insert competing interests here] |  |
| --- | --- |
| * typeset |  |
| Ethics Statement  Enter an ethics statement for this submission. This statement is required if the study involved:   - Human participants - Human specimens or tissue - Vertebrate animals or cephalopods - Vertebrate embryos or tissues - Field research   Write "N/A" if the submission does not require an ethics statement.  General guidance is provided below. Consult the [submission guidelines](http://journals.plos.org/plosone/s/submission-guidelines#loc-guidelines-for-specific-study-types) for detailed instructions. Make sure that all information entered here is included in the Methods section of the manuscript. | N/A |

Format for specific study types

Human Subject Research (involving human

participants and/or tissue)

Give the name of the institutional review

board or ethics committee that approved the

study

•

Include the approval number and/or a

statement indicating approval of this

research

•

Indicate the form of consent obtained

(

written/oral) or the reason that consent was

not obtained (e.g. the data were analyzed

anonymously)

•

Animal Research (involving vertebrate

animals, embryos or tissues)

Provide the name of the Institutional Animal

Care and Use Committee (IACUC) or other

relevant ethics board that reviewed the

study protocol, and indicate whether they

approved this research or granted a formal

waiver of ethical approval

•

Include an approval number if one was

obtained

•

If the study involved

non-human primates

,

add

additional details

about animal welfare

and steps taken to ameliorate suffering

•

If anesthesia, euthanasia, or any kind of

animal sacrifice is part of the study, include

briefly which substances and/or methods

were applied

•

Field Research

Include the following details if this study

involves the collection of plant, animal, or

other materials from a natural setting:

Field permit number

•

Name of the institution or relevant body that

granted permission

•

Data Availability

Authors are required to make all data

underlying the findings described fully

available, without restriction, and from the

time of publication. PLOS allows rare

exceptions to address legal and ethical

concerns. See the

[PLOS Data Polic](http://journals.plos.org/plosone/s/data-availability)

[y](http://journals.plos.org/plosone/s/data-availability)

and

[FA](http://journals.plos.org/plosone/s/data-availability#loc-faqs-for-data-policy)

[Q](http://journals.plos.org/plosone/s/data-availability#loc-faqs-for-data-policy)

for detailed information.

Yes - all data are fully available without restriction

| A Data Availability Statement describing where the data can be found is required at submission. Your answers to this question constitute the Data Availability Statement and will be published in the article, if accepted. |  |
| --- | --- |
| Important: Stating ‘data available on request from the author’ is not sufficient. If your data are only available upon request, select ‘No’ for the first question and explain your exceptional situation in the text box. |  |
| Do the authors confirm that all data underlying the findings described in their manuscript are fully available without restriction? |  |
| Describe where the data may be found in full sentences. If you are copying our sample text, replace any instances of XXX with the appropriate details.   - If the data are held or will be held in a public repository, include URLs, accession numbers or DOIs. If this information will only be available after acceptance, indicate this by ticking the box below. For example: All XXX files   are available from the XXX database (accession number(s) XXX, XXX.).   - If the data are all contained within the manuscript and/or Supporting Information files, enter the following: All relevant data are within the manuscript and its Supporting Information files. - If neither of these applies but you are able to provide details of access elsewhere, with or without limitations, please do so. For example:   Data cannot be shared publicly because of [XXX]. Data are available from the XXX Institutional Data Access / Ethics Committee (contact via XXX) for researchers who meet the criteria for access to confidential data.  The data underlying the results presented in the study are available from (include the name of the third party | All relevant data are within the manuscript and its Supporting Information files. |

| and contact information or URL).  • This text is appropriate if the data are owned by a third party and authors do not have permission to share the data.  * typeset |  |
| --- | --- |
| Additional data availability information: | Tick here if the URLs/accession numbers/DOIs will be available only after acceptance of the manuscript for publication so that we can ensure their inclusion before publication. |

manuscript	[Click here to access/download;Manuscript;Dache et al.docx](https://www.editorialmanager.com/pone/download.aspx?id=35525794&guid=f11f8619-91c7-42fe-9391-c5f9dd090d4f&scheme=1)

1	**Benthic Foraminifera as bio-indicators of natural and**

# 2 anthropogenic conditions in Roscoff Aber Bay (Brittany, France) 3

4	Edwin Daché^1^, Pierre-Antoine Dessandier^1^, Ranju Radhakrishnan^1^, Valentin Foulon^2^, 5	Loïc Michel^3^, Colomban de Vargas^4^, Jozée Sarrazin^1^, Daniela Zeppilli^1^.

6

7 ^1^ Univ Brest, Ifremer, BEEP, F-29280 Plouzané, France
8^2^ ENIB - École Nationale d'Ingénieurs de Brest (945 avenue du Technopole - 29280
9 Plouzané - France)
10 ^3^ Laboratory of Oceanology, Freshwater, and Oceanic Sciences Unit of reSearch 11 (FOCUS), University of Liège, Allée du Six Août 13, 4000, Liège, Belgium
12 ^4^ Sorbonne Université, CNRS, Station Biologique de Roscoff, AD2M, UMR 7144,
13 ECOMAP, Roscoff, France
14 * Corresponding author
15 E-mail: Edwin.Dache@ifremer.fr (ED)

16

17

18	**Key words**: benthic foraminifera, bioindicators, seasonal effect, anthropic impact, 19	pollution monitoring

# Abstract

Living benthic foraminifera, good environmental bio-indicators of both natural and anthropogenic conditions in marine environments, were investigated at a coastal environment in the Roscoff Aber Bay (Brittany, France).Eight sampling sites subjected to natural variations (freshwater inputs, tides) and/or anthropogenic impacts (pollution) were studied over four seasons in 2021-2022 (November, February, May, August). The main objective of this work is to analyse the spatial distribution of foraminiferal populations within and between sampling sites during different seasons and identify opportunistic species and those tolerant to anthropogenic impacts. To this end, sedimentology and biogeochemisitry of the sediments were examined by measuring grain size, temperature, oxygen, salinity, pH, pigment concentration (chlorophyll *a* and phaeopigments), total organic carbon (TOC), as well as the isotopic ratio of carbon (δ^13^C), nitrogen (δ^15^N) and sulfide (δ^34^S). Using these data as potential driving factors, four environments were distinguished among the sampling sites: open water, terrestrial, oligotrophic, and eutrophic, showing an increasing gradient of organic supply as well as very different microbial activities highlighted by carbon and sulphide isotopic ratios. The lowest abundance but highest diversity of foraminifera in the harbour is associated with the dominance of *Haynesina germanica* suggesting its particular tolerance to anthropogenic impacts. In the open water, *Ammonia beccarii* and *Elphidium crispum* were the most abundant species while *Quinqueloculina seminula* was the dominant species in the terrestrial environment. These preliminary data show an organic enrichment in the harbour due to anthropogenic activities that do not seem to significantly affect foraminiferal diversity. Benthic species in the Roscoff bay are good proxies for the marine environment conditions under various natural and anthropogenic conditions.

# Introduction

Foraminifera dominate the meiobenthos, being the most diverse and abundant group of shelled organisms in the marine environment [1]. They play a major role in organic matter cycling [2,3]. Their short life cycles and large spatial distribution make them sensitive to changes in environmental conditions as they respond rapidly to both natural and anthropogenic changes [4,5]. These

characteristics make them bioindicators for environmental quality or proxies to monitor environmental changes [4]. Several studies have shown that foraminifera are great model organisms to study anthropogenic impacts in coastal areas, such as pollutions in harbour [6], organic matter accumulation [7], thermal pollution [8] or oil accidents [9]. The Foram-AMBI index [10] has been used to identify groups of species from non-sensitive to tolerant and opportunistic species. Based on the standardized protocol established in [year?] by the FOBIMO group [11], the response of foraminiferal species in different study areas, such as in the Mediterranean Sea [12], Arctic fjords [10], intertidal zones [13] and estuaries [14], were identified.

Environmental changes in marine systems are challenging to monitor, especially because numerous factors come into play. In fact, the intertidal environments are impacted by both natural (e.g., tidal regime, meteorological and hydrodynamic variations, salinity gradients, temperature changes, sediment types, chemistry) and anthropogenic (e.g. eutrophication, contamination of heavy metals and chemicals, oil pollution and thermal impacts of power plants) stresses [4,15] making the evaluation of human impacts difficult to disentangle [16]. In addition, intertidal areas are characterized by a strong seasonal variability that is often ignored due to the analytical effort required to assess its evaluation. Complementary studies for environmental monitoring based on indices, including a more comprehensive dataset and considering seasonal variations are required in these environments. Among them, the concentration of organic carbon is one of the most important asIt is widely used as a proxy for eutrophication, often associated with the accumulation of pollutants and contaminants in coastal areas [10].

The natural variability of environmental conditions in the Roscoff Aber Bay, located in the French northwest coastal area, has been widely studied [17–19]. The bay is a large flat-bottomed depression located entirely above the mid-tide line [20]. It is characterized by habitats with very different grain sizes, strong variations in sea height due to tidal influence and inputs of fresh water, which affect its salinity. A channel is also used extensively by boats going to the nearby Batz Island. A harbour in the bay is impacted by inputs of organic matter linked to fishing and hydrocarbon spills. The Bay is subject to large tidal ranges as well as winter storms and has a diversity of highly heterogeneous habitats [21]. Two monitoring stations (one coastal, the other littoral) collect data on numerous environmental and biological parameters [22], thus providing descriptions of environmental conditions in this dynamic environment.

The Roscoff biological station, has monitored coastal plankton [23,24], algae [25–27], meiofaunal distribution and trophic ecology of nematodes [31], and macro-organisms [28–30], indicating high biodiversity and habitat variety. . However, this area is also characterized by human influence such as tourism and fishing activities for which the impact on the meiobenthos remain poorly described. Monitoring benthic foraminifera has never been carried out in this area. Their use as bio-indicators could answer questions about human impacts: Does the Roscoff area foraminiferan community responds to anthropogenic impacts and can we discriminate this response from natural changes? Is there a seasonal response? Can we use bio-indicator benthic foraminiferal species to discriminate the different environmental changes that occurred in this area?

To answer these, we analysed the spatial and temporal distribution of living benthic foraminifera from eight habitats impacted by natural and anthropogenic changes in order to identify bio-indicator species. To assess the seasonal and spatial variabilities, sampling was conducted in November, February, May, and August at eight sites in the inner and outer part of the bay as well as in the harbour. We focus on the distribution and abundance of living foraminiferal species, aiming at determining their ecology and identifying species that are sensitive and tolerant of the changes. Ultimately, we will compare our results with existing ecological indices. Furthermore, fluorescence has been used to observe the presence of potential kleptoplasty on the foraminiferal species *Haynesina germanica* [32,33].

# Materials and Methods

## Study area

The study area is located on the coast of Roscoff (Brittany, France), within a small bay of 2 km^2^. The bay consists of a small 2 km-long and 1 km-wide cove, partly silted up with different types of intertidal sediments [34]. A freshwater stream which communicates between the polder and the sandy-muddy north-eastern region enters the cove. The cove is very shallow, located completely above the mean tide level, with strong currents and a tidal range of about 4 meters, allowing complete mixing and high turbidity [20]. A series of eight sampling sites, differently subjected to anthropogenic impacts, were selected. Stations 1-5 are located in the subtidal zone of the bay, station 5 being nearest to a freshwater stream (Fig 1). Station 6 is located in the old fishing port of Roscoff (Fig 1). Stations 7 and 8 are subtidal zones located outside the bay and subject to strong channel currents between Batz Island and Roscoff (Fig 1). Environmental and faunal sampling were done in November 2021, February 2022, May 2022, and August 2022.

**Fig 1. This is the Fig 1 Satellite map showing the position of the different sampling stations on the Roscoff Aber Bay.** This is the Fig 1 Stations 1-5 in the Bay, station 6 in the harbour and stations 7-8 outside of the Bay.

## Sample collection

30 cm x 30 cm quadrats were deployed at each of the eight station and a Plexiglas corer of 3 cm diameter and 5 cm height, representing a volume of 35 cm³ was used for sampling [35]. Three cores per station were taken within the quadrat to analyse granulometric, pigmentary and isotopic parameters, for a total of 24 cores per season. These cores were frozen at -20°C. Three sediment cores per station were also taken for subsequent analysis of the associated meiofauna. In this study, only a single core will be used for foraminiferal determination. The sediment cores were first immersed in 6% MgCl_2_ for 10 minutes before fixation with 4% borax buffered formalin in order to extract the meiofauna from the sediments by centrifugation with LUDOX® colloidal silica [35]. To help with data analysis, a SOMLIT (Service d'Observation en Milieu Littoral) sampling station located near our study area provided regular data on environmental parameters [36]. We were able to plot salinity, oxygen, pH and Chlorophyll a data for the period of November 2021 to August 2022 (S 1, Fig 1).

**S 1 Fig 1. This is the S 1 Fig 1 SOMLIT data for salinity, oxygen, pH and Chl *a*.** This is the S 1 Fig 1. SOMLIT data for salinity, oxygen, pH and Chl *a* over the sampling period from November 2021 to August 2022 in the ESTACADE Somlit sampling point. The data was extracted from the SOMLIT database (Service d'Observation en Milieu Littoral; www.somlit.fr) on 5 October 2022.

## Sedimentary and geochemical analyses

### Grain size analysis

Particle size analysis was carried out with a Malvern™ Mastersizer 3000 laser diffractometer which has a measurement range of 0.01- 2200 µm. The measurements were performed on the entire sediment core. In this study, only the statistical mode Q 50 is represented, the average particle size of each sampling station [37].

### Oxygen, pH, temperature, and salinity measurement

At all stations, salinity parameters were measured with the LF 340 handheld conductivity meter with standard TetraCon 325 conductivity cell and pH parameters were measured with the WTW pH 3310 sensor. Temperature and oxygen measurements were made with the Oxygen Optode 3830. Measurements were taken directly during core sampling in a hole in the sediment with interstitial seawater. Once the measurement had stabilised, the maximum value was taken. This procedure was repeated throughout the seasons.

### Pigment analysis

Photosynthetic pigments were analysed for the entire sediment core. The pigments were extracted with 99.9% methanol solvent. The supernatant was centrifuged several times to avoid sediment in the HPLC system. 170 μL of the extract and 30 μL of water were mixed and a 100 μL aliquot of this mixture was immediately injected into a high-performance liquid chromatography (HPLC) system. Filtration, extraction procedures and HPLC pigment analyses were performed following Zapata *et al.* [38]. Total organic carbon (TOC) and Chlorophyll a (Chl *a*) indicate the total amount of Organic Matter (OM) and phytodetritus, while the quality of OM is assessed using the ratio of Chl *a*/Phaeopigments (Chl *a*/Phaeo), which indicates the freshness of the phytodetrital material [37].

### Elemental and isotopic analyses

Sediment samples were freeze-dried at -50°C and ground to a fine powder using a grinder. Sediment aliquots were ground to a homogeneous powder using mortar and pestle and acidified to remove carbonates by direct addition of excess 1M HCl in small increments, and subsequently rinsed with distilled water. Sediment samples were analysed twice: once using acidified material (for total organic carbon content and carbon stable isotope ratios) and once using native material (for total carbon content, total nitrogen and sulphur content and nitrogen and sulphur stable isotope ratios).

Elemental content was measured using a vario MICRO cube C-N-S elemental analyser (Elementar Analysensysteme GMBH, Hanau, Germany) as relative percentage of analysed mass (mass%). Empty tin cups were used as analytical blanks. Sulfanilic acid (Sigma-Aldrich; %C = 41.6%, %N = 8.1%, %S = 18.5%) was used as the elemental standard.

Stable isotope ratio measurements were performed via continuous flow - elemental analysis - isotope ratio mass spectrometry (CF-EA-IRMS) at University of Liège (Belgium), using the abovementioned vario MICRO cube C-N-S elemental analyser coupled to an IsoPrime100 isotope ratio mass spectrometer (Isoprime, Cheadle, United Kingdom). Isotopic ratios were expressed using the conventional δ notation [39], in ‰ and relative to the international references Vienna Pee Dee Belemnite (for carbon), Atmospheric Air (for nitrogen) and Vienna Canyon Diablo Troilite (for sulphur). IAEA (International Atomic Energy Agency, Vienna, Austria) certified reference materials sucrose (IAEA-C-6; δ^13^C = -10.8 ± 0.5‰; mean ± SD), ammonium sulphate (IAEA-N-2; δ^15^N = 20.3 ± 0.2‰; mean ± SD), and silver sulphide (IAEA-S-1; δ^34^S = -0.3‰) were used as primary analytical standards. Sulfanilic acid

(Sigma-Aldrich; δ^13^C = -25.6 ± 0.4‰; δ^15^N = -0.13 ± 0.4‰; δ^34^S = 5.9 ± 0.5‰; means ± SD) was used as a secondary analytical standard. Standard deviations on multibatch replicate measurements of secondary and internal lab standards (coastal Mediterranean sediments) analysed interspersed with samples (one replicate of each standard every 15 analyses) were 0.2‰ for both δ^13^C and δ^15^N and 0.5‰ for δ^34^S.

### Benthic foraminiferal faunal abundance and diversity

The eight benthic foraminiferal samples per season were preserved in 4% formalin with phloxine B to stain the endoplasm of live benthic foraminifera. The samples were wet sieved through 20, 100 and 1000 μm mesh sizes. Only the fraction > 100 μm was used for this study. Samples were sorted by hand under a binocular microscope and stained benthic foraminifera were collected and preserved on micropalaeontological slides. The faunal densities for each layer were standardised for a sediment volume of 35 cm³. The species richness (S) corresponding to the observed number of species present in a sample, the Shannon index H′ [40] for species diversity and the evenness E as eH′/S of Buzas and Gibson, 1969 [41] were used to compare the diversity of species between samples. PAST software was used to calculate these different indices [42]. Rarefaction curves were also drawn for each station to evaluate the completeness of our sampling approach. Species representing less than 5% were grouped together in the "other" category.

### Foraminifera fluorescence

*Haynesina germanica* and *Quinqueloculina seminula* from all samples previously manually isolated and sorted by species on microslides (plummer cell) were imaged. A motorized binocular Zeiss AxioZoom V16 equiped with a HXP-120 light source and a Plan NeoFluar 1.0X was used at 20X total magnification (pixel size =

5.16µm). Brighfield and red fluorescence channels (Ex 559-585nm, Em 600-690nm, BeamSplitter 590nm) were imaged with a AxioCam HR R3. Red fluorescence channel covers the chlorophyll autofluorescence spectra. Thus, images were processed on Fiji [43] to measure the sum of the value of pixels of the fluorescence channel and the relative fluorescence area of individual *H. germanica* and *Q. seminula*. Briefly, image mask was automatically generated by Huang threshold on brightfield channel to isolate each specimen to one ROI (region of interest). Otsu threshold was used in each previous ROI on the fluorescent channel before measurement of RawIntDen (the sum of all pixel values in the ROI) and %area.

### Statistical analyses

Environmental variables were first subjected to principal components analysis (PCA) to visualize their spatio-temporal distribution. In order to characterize the distribution of the most dominant species in the area in relation to environmental conditions, a redundancy analysis (RDA) (Fig. 7) was performed, and an ANOVA test was used to evaluate the significance of the RDA results [44] . After selecting the environmental variables (temperature, TOC, TC, grain size, δ^34^S and oxygen) for the RDA, the adjusted R² value was calculated to discriminate the environmental variables that explain most of the variance in species abundance. The ANOVA test produced a p-value for the model equal to 0.001, confirming that the model is statistically

significant.

# Results

## Environmental conditions within the sediments

### Temperature, pH and salinity

Temperatures recorded during sampling showed a seasonal variability, with a difference of 2°C for February between November and May and 8°C with August. The difference between stations remains very slight for November, February and May, but for August, the temperatures in stations 1, 2 and 7 varied between 21 and 22°C, compared to 28°C for station 6 (Table 1).

pH varied very little between seasons and between sites. Values ranged from 7.2 to 8.8. In terms of salinity, a few drops were observed, particularly in November at station 3 (14.9) and in May and August at station 5 (15.6 and 10.3) (Table 1).

**Table 1. This is the Table 1 Environmental parameters in the Bay of Roscoff.** This is the Table 1 Environmental parameters characterizing the eight sampling stations during the four seasons of the study in the Bay of Roscoff (2021-2022).

### Grain sizes

Grain size did not change between seasons, except for station 8 with a Q50 of 906 µm for November and 250 µm for May. Except for the coarse station 8, all stations had fine to medium sand grains ranging from 168 to 371 µm. Station 6 had the finest sediment, varying between 102 and 125 µm.

## Geochemistry of sediments

### Oxygen concentrations

Very large differences in oxygen concentrations were observed between the sites over the four seasons. Oxygen concentration was highest in November, followed by May. Oxygen concentrations were lowest in February and August. In November, the highest concentrations were found at stations 3 and 7, and the lowest at stations 1, 5 and 6. In February, the highest concentrations were found at station 3 and the lowest at stations 2, 4, 5 and 6. In May, concentrations were highest at stations 2, 3 and 4 and lowest at stations 1, 5, 6 and 7. Very low concentrations were observed in August, the highest being at stations 3 and 4 and the lowest at stations 1, 5, 6 and 7. Seasonal variations of oxygen between stations showed that station 1 did not vary except for a decrease in August. Station 2 had high concentrations in November and May, but very low concentration in February and August (Table 1). Similarly, station 3 showed a decrease in oxygen concentration in August. Station 4, like station 2, has high concentrations in November and May but very low concentrations in February and August. Station 5 has extremely low concentrations in February, May and August at station 6, concentrations were low in November and August. Oxygen concentrations at stations 7 and 8 decreased from November to August. For station 7, May and August had very low concentrations. At station 8, concentrations in August were also very low (Table 1).

### Total organic carbon (TOC), Total Carbon (TC), Nitrogen, Sulphur, δ13C, δ15N, δ34S

Total carbon (TC) values ranged from 0.2 to 2.5 ‰. In all seasons, the stations with the highest TC were stations 2, 6 and 8. Station 7 also had high TC, but in February only. Variations in TC between seasons within other stations were minimal (Table 1).

Total organic carbon (TOC) values varied between 0.1 to 1.4 ‰, always reaching its maximum at station 6, with values 2 to 3 times higher than the others, up to 1.4 ‰. Station 5 in November, May and August and station 8 in November February and May had a slightly higher TOC than the other stations (Table 1).

Nitrogen content (%N) over all seasons was higher in station 6 but also in station 8 in November, February, and May. Station 7 also showed an increase in nitrogen content (~4 times higher) in February. %N in station 6 peaked in November and August (~2 times higher). Inter-season changes were limited in other stations (Table 1).

For the sulphur content (%S), it was higher at station 6 at all seasons and also, at station 5 in February. The changes in sulphur content (%S) between seasons were almost the same at all stations, with a gradual increase from November to August (Table 1).

For δ^13^C values, there were no great differences between November, February, and May. August had the highest δ^13^C values. Station 1 had the highest δ^13^C values and stations 5 and 6 the lowest.

In terms of δ^15^N values, station 3 had slightly higher overall values, and station 6 had slightly lower values (Table 1). The δ^34^S values show a consumption of the heavy isotope which caused the isotopic fragmentation at station 6 to decrease at all seasons. The same observation can be made at station 7 in February and at station 5 in May and August (Table 1).

### Pigments

The highest levels of Chlorophyll *a* (Chl *a*) were observed in May, mainly at stations 3, 5 and 8 (Table 1). Between the different stations and seasons, the quantities of Chl *a* were relatively heterogeneous. Station 3 had the highest quantity of Chl *a* accumulated over all seasons (192 µg Chl *a*/cm²), while station 6 had the lowest (63 µg Chl *a*/cm²). The lowest values were recorded in February at stations 6 and 8, and in November at station 3 (Table 1). The ratio of Phaeopigment to Chl *a* was higher in station 6, particularly in November, February, and May, with a maximum of

0.40 (Table 1). Some stations, such as station 3 in November, had a ratio of 0.29 (Table 1). Contrary to the Chl *a* at station 8 in February, which was characterized by high levels of phaeopigments.

CAN’T ALL THESE DESCRIPTIONS BE ELIMINATED BY SIMPLY REFERRING TO TABLE 1? IF THE DIFFERENCES MEAN ANYTHING LATER, THE TABLE MAY BE REFERRED TO.

### Foraminiferal densities and composition

The abundance of foraminifera between the different stations was heterogeneous. The lowest abundance of foraminifera was observed in May with a total for all stations of 2094 individuals/100 cm² while August showed the highest abundance with 23 696 ind./100 cm². For all seasons combined, station 5 had the lowest abundance with 1415 ind./100 cm² while the highest abundance was observed at station 2 with 15 463 ind./100 cm². Abundances within stations varied from a maximum of 6607 ind.100/cm² for station 2 in November to a minimum of 28 ind./100 cm-² for station 8 in May (Fig 2).

A total of 29 species were identified, of which 9 accounted for more than 5%

(Fig 2) these species were images with scanning electron microscope (Fig 3). *Quinqueloculina seminula* was the most abundant, particularly in November, and was dominant at station 2 at all seasons (Fig 2). Very low abundances were observed at stations 5, 6, 7 and 8 in February and at stations 3, 4 and 8 in August (Fig 2). *Elphidium crispum* was abundant at stations 7 and 8 in February and August and at station 1 in November (Fig 2). This species was absent from station 5 at all seasons. *Ammonia beccarii* was also abundant at stations 7 and 8 in February and August (Fig 2) but rarely observed at the other stations. *Haynesina germanica* was dominant at station 6, particularly in August, and abundant at station 2 also in August. Only a few occurrences were observed at station 4 in November (Fig 2). This species was absent from stations 1 and 8 throughout the four seasons. *Cibicides lobatulus* was particularly abundant at stations 1 and 2 in November, with maximum densities at station 3 in August. It was also present at stations 1, 2, 6, 7 and 8 in February, with a more homogeneous distribution (Fig 2). *Cribroelphidium gerthi*, was recurrently present at station 6, in higher abundance in August. Maximum abundance was also observed at station 4 in November (Table 1). *Reophax scorpiurus* was present at stations 2 and 6 in May, with a significant increase in abundance at stations 1, 4 and 5 in August (Table 1).

**Fig 2. This is the Fig 2 Abundance of the major living species of benthic foraminifera.** This is the Fig 2 Densities (expressed in number of specimens per 100 cm^2^) of the major (>5%) living species of benthic foraminifera in the Bay of Roscoff at the eight sampling stations and for the four months of the study.

**Fig 3. This is the Fig 3 Scanning electron micrographs of the species of benthic foraminifera in the Bay of Roscoff.** This is the Fig 3 SEM images of the species of foraminifera found in the various stations sampled in the Bay of Roscoff.

### Species diversity

Species richness varied from 1 at station 5 in February to 12 at station 6 in August (Fig 4 I). Over all seasons, stations 2 and 6 had more taxa while the lowest number of taxa was observed at station 5 (Fig 4). For most stations, May had the lowest number of taxa, 2.5 times lower than in August. The same number of species was observed in November and February (Fig 4). Station 5 had 3 to 5 species, but only one in February (Fig 4 F). Station 6 had a similar number of taxa in November and May and a maximum number of species in February and August (Fig 4).

**Fig 4. This is the Fig 4 ) Rarefaction curves and Shannon index, Evenness index and number of taxa.** This is the Fig 4 A) to D) Rarefaction curves for all stations from November 2021 to August 2022, E) to I) Shannon index H' (green), Evenness index E

(light blue) and number of taxa (dark blue) based on observed living individuals.

The Shannon index is the highest at station 6 over all seasons, with a maximum in February, and lowest at station 5, particularly in February due to the presence of a single species in the core (Fig 4). February and August have the highest Shannon index and May the lowest (Fig 4 F, H and I). For station 1, a 50% decrease occurs in May and August (Fig 4 H and I). The index remains constant at station 2 except for an increase in August (Fig 4 I). Stations 3 and 4 have a higher index in February and August (Fig 4 F and I). Station 6 showed a slight drop in this index in August compared to the other season (Fig 4 I). Stations 7 and 8 had constant indices for all seasons except for station 8, which had a drop in May (Fig 4 I).

The Evenness index (E) has the highest value at station 5 in February, as only one taxon and 5 individuals occur, followed by stations 1 and 8 in May (Fig 4 F and H). The E index was lowest at all stations in August (Fig 4 I). The lowest E index was observed at station 2 over all seasons (Fig 4). Stations 4 and 5 showed little difference in index in August, as opposed to other stations (Fig 4 I).

The rarefaction curves indicated that the sampling effort was sufficient, particularly at stations 2, 4 and 7 in November (Fig 4 A), stations 2, 6, 7 and 8 in

February (Fig 4 B), stations 2 and 6 in May (Fig 4 C) and stations 4 and 7 in August (Fig 4 D). For the other stations, the curves did not reach a plateau, indicating that more samples would be required to adequately describe the diversity. However, the low diversity observed over abundant samples in this coastal system indicates that a low number of species can be expected. In November, at station 2, the number of species was at a maximum and the curve flattened out at 10 taxa, which indicates that the number of samples was sufficient to estimate species richness (Fig 4 A). In February (Fig 4 B), at stations 2 and 6, the number of species was at its maximum and the curve flattened out at between 9 and 10 taxa. In May (Fig 4 C), at stations 2 and 6, the number of species was highest, and the curve flattened out at 5 and 6 taxa. In August (Fig 4 D), at station 6, the number of species was highest, and the curve flattened out at 12 taxa, followed closely by stations 2, 3 and 8 at 11 taxa.

### Foraminiferal fluorescence

To confirm the presence of a phytoplankton efflorescence, which was not visible in the HPLC data, two species of foraminifera from different seasons were scanned with the Axiozoom using the fluorescence of chlorophyll and organic matter. The primary aim of this experiment was to demonstrate the kleptoplasticity of *H. germanica* compared with the non-kleptoplastic *Q. seminula*. This fluorescence can be seen in S 3 Fig 3. Fluorescence was observed in the two species of foraminifera throughout the seasons (Fig 5). Fluorescence intensity in August was low for both species (Fig 5 C and D). A high fluorescence intensity was observed in May for *H. germanica*, which was higher than at the other seasons (Fig 5 C). However, the area of fluorescence in May did not increase significantly compared to the intensity of fluorescence over this period (Fig 5 A and B).

**S 3 Fig 3. This is the S 3 Fig 3 Plate of *Haneysina Germanica* foraminifera to compare brighfield and red fluorescence channel.** This is the S 3 Fig 3. Plate of *Haneysina Germanica* foraminifera at station 4 in November 2022 in brighfield (A) and red fluorescence channel (Ex 559-585nm, Em 600-690nm, BeamSplitter 590nm) (B) and Illustration of a density plot for red fluorescence (C).

**Fig 5. This is the Fig 5 Percentage of fluorescence and fluorescence intensity for *Haynesina germanica* and *Quinqueloculina seminula*.** This is the Fig 5. Percentage of fluorescence air for (A) *Haynesina germanica* and (B) *Quinqueloculina seminula*) as well as fluorescence intensity for (C) *Haynesina germanica* and (D)

*Quinqueloculina seminula*).

# Discussion

## Microhabitats

Benthic foraminifera are mainly dependent on the availability of food and oxygenation of the ecosystem in which they live, as shown by the TROX model [45]. In very oligotrophic environments, shallow infaunal species, adapted to a low trophic level, thrive close to the sediment-water interface in a well-oxygenated environment. In more eutrophic conditions, they are rather restricted to the first few millimeters of sediment, having only limited tolerance to low oxygen conditions [46]. Sediment grain size and the quantity and quality of natural organic matter are factors that control faunal distribution [47,48]. In estuarine environments, foraminiferal species biodiversity is low [49], with a dominance of species adapted to variable conditions. In fact, the spatial and temporal variations in physical and chemical parameters are high, potentially inducing natural stress (e.g., salinity, temperature, water movement, turbidity, oxygen content, organic matter inputs, tidal cycle, etc.).

The bay of Roscoff has very diverse habitats. The stations were chosen to provide a range of environmental conditions over a small area, taking natural and anthropogenic influences into account. They include freshwater inputs, harbour conditions, tide levels and channel influence. Stations 1 to 5 are located in the bay, with freshwater inflows influencing stations 3 and 5 in particular [50]. Deposits of green algae due to the ephemeral proliferation of *Enteromorpha* spp. are also present at these stations [51], linked to major inputs of nutrients [52]. A freshwater outlet is also present at station 4, which is situated close to a thalassotherapy centre. Station 6, located in the harbour, is influenced by fishing waste discharged the water where it accumulates and decomposes, resulting in a high concentration of organic matter, as shown by the TOC data for this station [21]. Stations 7 and 8 are located outside the bay, close to the channel linking Roscoff and Batz Island. They are less sheltered from the marine contributions and therefore could be more importantly affected by currents.

## Environmental conditions

### Physico-chemical domains

The PCA of environmental data shows that axis 1 explains 34.85% of the variability (Fig 6). Nitrogen, sulphur, and TOC parameters are positively loaded on PC1 and correlated with station 6 (harbour), representing the eutrophic domain (Fig 6). Oxygen, δ^34^S, δ^15^N and δ^13^C, associated with stations 1, 2, 3 and 4 are negatively loaded on PC1, hence representing the oligotrophic domain. δ^34^S and δ^13^C are lower in the eutrophic domain, respectively reflecting a higher isotope fragmentation of sulphur and carbon in the harbour probably caused by high biological activity (Table 1). PC2 explains 15.17% of the environmental variability (Fig 6). On this axis, grain size, salinity, Chl *a* and TC are positively loaded, associated with stations 7 and 8, highlighting the dynamic context of this open water system. pH and temperature are negatively loaded on PC2, associated with the stations located within the Roscoff Aber Bay (2, 3, 4 and 5), suggesting a more terrestrial domain. The phaeopigments are higher in the eutrophic zone due to the degradation of Chl *a* in the port and terrestrial zones.

**Fig 6. This is the Fig 6 Principal component analysis based on the environmental parameters.** This is the Fig 6 Principal component analysis based on the environmental parameters (Grain size, Oxygen (O), pH, Temperature (T°C), salinity, Chlorophyll *a* (Chl *a*), phaeopigments, Total organic carbon (TOC), Total

Carbon (TC), Nitrogen (N), Sulphur (S), δ^13^C, δ^15^N, δ^34^S) sampled during four seasons at the eight sampling stations.

### Seasonality

The temperatures rose at all stations with the onset of summer.

Salinity remains constant over time, except a few notable decreasing events in November at station 3 and in May and August at station 5. These stations are close to a freshwater outlet, which could explain lower salinities, particularly in November when rainfalls occurred from mid-October to early November (S 2 Fig 2). However, the drop in salinity in May and August did not follow particularly high rainfall and would likely be due to resurgence observed in this zone influenced by freshwater outflow from the bottom of the Aber [50]. Somlit data show that the spring bloom began at the end of March, as can be seen by the concentrations of Chl *a* (~ 1 µg. L^-1^ and rising to ~ 4 µg. L^-1^) (S 1 Fig 1 D). The influx of freshwater resulting from precipitation between March 30 and April 13 most likely provided terrestrial nutrients, triggering a spring phytoplankton bloom. This episode of high primary production ended in late May to early June. Consequently, the samples collected at the beginning of May 2022 were taken right in the middle of this bloom.

This temporary condition could explain the relatively moderate contribution of marine OM in the surface sediments. In contrast, HPLC analyses only show an increase in Chl *a,* but not in marine detritus at station 3, indicating a terrestrial origin of chlorophyll at this station. These dissociated signals in the water column and sediments could be explained by the time lag between the bloom period and phytoplankton arrival to the seafloor.

**S 2 Fig 2. This is the S 2 Fig 2 Rainfall data for Morlaix.** This is the S 2 Fig 2.

Rainfall data for Morlaix (21 km of Roscoff Aber Bay in Brittany, France) for the period October 2021 to August 2022 from the infoclimat.fr/climatologie/globale/31aout/morlaix/000AW.html website.

The first hypothesis explaining the low faunal abundance in May is that the samples were taken too soon after the bloom and that the organic matter was degraded throughout the year before being renewed in the spring. The sampling would then be synchronous with the start of the spring cohort of foraminifera after an episode of natural mortality. The second hypothesis is that storms, with strong wind gusts, may have periodically disturbed the ecosystem, resulting in the washing of sediments in May, which would have decreased OM and may have caused the transport of organisms. The third hypothesis is that there may be competition between foraminifera and other meiofaunal taxa (nematodes, copepods, etc.).

## Spatio-temporal distribution of foraminifera

The RDA analysis indicates that the more dynamic subtidal zone is

characterised by a dominance of *A. beccarii* and *E. crispium* (Fig 7) that prefer more marine conditions [49]. *Ammonia beccarii* have been shown to be tolerant to variations in substratum types and total organic carbon [53]. In the oligotrophic habitat, *Q. seminula* is dominant, associated with higher oxygen concentrations (Fig 7), confirming its prevalence in well-oxygenated environments [54], as particularly observed at station 2. This species is associated with areas with high hydrodynamics and has a preference for marine conditions [49,55]. It can also feed on phytodetritus and is probably capable of rapidly ingesting fresh organic matter from marine primary production [56,57]. The eutrophic environment is dominated by *H. germanica* and *C. gerthi*, associated with high TOC and temperatures (Fig 7). *Cribroelphidium gerthi* is also abundant at station 6, being able to live in low-oxygen environments [58] throughout all seasons. *Haynesina germanica* has been identified as a bioindicator of pollution correlated with anthropogenic activities, such as pollutants introduced by industrial activity [59,60]. Despite their short reproductive cycles and high reactivity to environmental changes, a time lag of two to four weeks is required for a foraminiferal response [14].

**Fig 7. This is the Fig 7 Redundancy analysis of foraminiferal species**. This is the Fig 7. Redundancy analysis (RDA) of foraminiferal species *(Ammonia beccarii, Elphidium crispum, Elphidium aculeatum, Cibicides lobatulus, Cribroelphidium gerthi, Haynesina germanica, Quinqueloculina seminula, Quinqueloculina oblonga, Reophax scorpiurus)* (Hellinger-transformed abundance) with environmental variables (Grain Size, Total Carbon (TC), Temperature (T°C), Total organic carbon (TOC), Oxygen (O), δ^34^S, pH), P < 0.05).

However, although *H. germanica* clearly shows a fluorescence increase in May, this phenomenon was not observed in *Q. seminula*. This increase could be either due to a phytoplankton bloom allowing a supply of fresh organic matter or to its capacity to perform kleptoplasty, therefore keeping chloroplasts for later use [32,33]. The absence of signal in *Q. seminula* rather points for the second hypothesis. This process would explain its abundance in August at 5 stations (st 2, 3, 4, 6 and 7).

This suggests that the May samples were taken during the early phase of the bloom with non-degraded diatoms, shortly later ingested by a new generation of fauna. These environments are often linked with increased pollution by heavy metals, which can lead to a reduction in the abundance and diversity of foraminifera [61], as well as the development of calcareous shell deformations [62]. A double aperture was observed in *Bolivina* sp. in August at station 6 (S 4 Fig 4). This season of higher temperatures may have impacted foraminifera growth, resulting in test abnormalities, as reported in polluted areas [4]. However, station 6 remains diverse compared with the other sampling stations. This could indicate eutrophication resulting from fishing wastes, rather than pollution affecting biodiversity. In fact, in estuaries natural characteristics stress may be similar to those of anthropogenic activities. This observation is known as the «estuarine paradox» and indictes that environmental indicators should be used carefully.

[16,49,63].

**S 4 Fig 4. This is the S 4 Fig 4 Scanning electron micrographs of *Bolivina sp***. This is the S 4 Fig 4. SEM image of *Bolivina sp.* in August 2022 at station 6 with a morphological anomaly of a double aperture.

The high abundance of *Cibicides lobatulus* in August at station 3, which was not present during the other seasons, could be explained by the fact that this species lives attached to algae [64]. These specimens may have been transported as a result of the heavy rainfall from late June to early July (S 2 Fig 2) and freshwater resurgence that were responsible for the export of terrestrial matter to the marine system [65]. *Reophax scorpiurus* also occurred in May. Subsequently, this species was abundant in August at stations 1, 4 and 5 in the intertidal zone. On the RDA, this species is strongly correlated with temperature and TOC (Fig 7). The presence of agglutinants (*Reophax* genus) that are tolerant to a variety of physicochemical conditions [57] may be explained by drastic temperature increase and eutrophication conditions. These species could have been pioneer recolonizers after an event in May that affected the other species and opened new niches. They have been observed in highly variable trophic conditions and seem able to tolerate variable OM quality [66,67]. We can postulate that this species is more competitive when the organic matter present is more refractory. Their increased occurrences in August suggest a response to a disruption in the ecosystem in May that opened new niches for pioneer recolonizers before summer conditions, favouring their establishment in the area.

# Summary and Conclusion

In this study, we designed an extensive environmental characterization at a seasonal scale in order to evaluate the response of the benthic foraminiferal fauna in a dynamic coastal area under anthropogenic influence. Several parameters influence this coastal environment among which salinity is considered as a dominant factor, but other controlling factors, such as the introduction of organic matter (marine and/or continental), temperature and the tidal cycle. The abundance of *A. beccarii* and *E. crispum* in the open water domain highlights their preference for larger grain sizes (Fig 8). Their stations are influenced by variations in salinity and Chl *a*, signs of a more heterogeneous habitat. As the marine contribution reduces, the foraminiferal fauna is influenced by continental inputs, resulting in a mixture of marine and terrestrial conditions, as shown within the bay with stations 2, 3, 4 and 5 correlated with pH, temperature, and oxygen. *Quinqueloculina seminula* is typical for this terrestrial and oligotrophic are a as well as *R. scorpiurus*, which occasionally takes advantage of a natural event of ecological stress to establish (Fig 8). Stations 3 and 5 are characterised by low foraminiferal abundance due to more stressful and less stable environmental conditions caused by the influx of fresh water from the river Aber. This study also highlights the eutrophic nature of the harbour area, influenced by higher concentrations of organic matter and sulphur, and characterised by the presence of *H. germanica* and *C. gerthi* (Fig 8). The Roscoff coastal zone shows that foraminifera are good indicators, for characterising the seasonal dynamic of the environment and can be used to bio-monitor events that impact the ecosystem, such as phytoplankton blooms, OM accumulation and potential human-derived stress. In the future, it would be interesting to set up monitoring over shorter periods, enabling the observation of more events, thanks to the short generation time (some weeks) of foraminifera.

**Fig 8. This is the Fig 8 Synthetic schema of major species distribution and abundance in Roscoff Aber Bay over the 4 seasons.** This is the Fig 8. Synthetic schema of major species distribution and abundance in Roscoff Aber Bay over the 4 seasons *(Ammonia beccarii, Elphidium crispum, Cibicides lobatulus, Cribroelphidium gerthi, Haynesina germanica, Quinqueloculina seminula, Quinqueloculina oblonga, Reophax scorpiurus)* with environmental variation (oxygen concentration, organic matters and fresh water).

## Authors’ contributions

E.D conceptualised, acquired most of the data set and led the writing. PAD supervised the work of E.D, acquired data and participated in the writing. RR acquired data. VF acquired data, took part in the sampling and contributed to the drafting. L M acquired data. CV participated in the supervision and administration of the project. JS and DZ supervised the work of E.D and participated in writing the publication.

# Acknowledgments

The authors would like to thank Sarah Garric and Christophe Six for their help and support with the HPLC analysis of the sediment pigments and the interpretation of the various dendrograms. The authors would like to thank Valentine Foulquier for her help with the field collection, extraction, and technical analysis of the samples. The authors would like to thank the SOMLIT platform for providing temporal data from November 2021 to August 2022 for the ESTACADE point. The authors would like to thank Nicolas Gayet for his help in acquiring SEM images of the foraminifera. The authors would like to thank Victor Simon for the loan of temperature, pH and salinity probes. The authors would like to thank Christelle Simon Colin for the loan of the freeze-drying machine. The authors would like to thank the scientific managers of the ESTACADE stations and the SOMLIT coordinator.

The authors declare that they have no conflicts of interests.

# References

1.	Balsamo M, Semprucci F, Frontalini F, Coccioni R. Meiofauna as a Tool for

Marine Ecosystem Biomonitoring. In: Cruzado A, editor. Marine Ecosystems. InTech;

2012. doi:10.5772/34423

2. Altenbach A, Sarnthein M. Productivity Record in Benthic. 1989.
3. Gooday AJ, Levin LA, Linke P, Heeger T. The role of benthic foraminifera in deep-sea food webs and carbon cycling. Deep-sea food chains and the global carbon cycle. 1992; 63–91.
4. Alve E. Benthic foraminiferal responses to estuarine pollution; a review. The

Journal of Foraminiferal Research. 1995;25: 190–203. doi:10.2113/gsjfr.25.3.190

5. Vassallo P, Fabiano M, Vezzulli L, Sandulli R, Marques J, Jorgensen S. Assessing the health of coastal marine ecosystems: A holistic approach based on sediment micro and meio-benthic measures. Ecological Indicators. 2006;6: 525–542. doi:10.1016/j.ecolind.2005.07.003
6. Du Châtelet EA, Gebhardt K, Langer MR. Coastal pollution monitoring:

Foraminifera as tracers of environmental perturbation in the port of Boulogne-sur-Mer

(Northern France). njgpa. REFEREMCE? 2011;262: 91–116. doi:10.1127/0077-7749/2011/0187

7. Mojtahid M, Jorissen F, Pearson TH. Comparison of benthic foraminiferal and macrofaunal responses to organic pollution in the Firth of Clyde (Scotland). Marine

Pollution Bulletin. 2008;56: 42–76. doi:10.1016/j.marpolbul.2007.08.018

8. Arieli RN, Almogi-Labin A, Abramovich S, Herut B. The effect of thermal pollution on benthic foraminiferal assemblages in the Mediterranean shoreface adjacent to Hadera power plant (Israel). Marine Pollution Bulletin. 2011;62: 1002–1012. doi:10.1016/j.marpolbul.2011.02.036
9. Ernst SR, Morvan J, Geslin E, Le Bihan A, Jorissen FJ. Benthic foraminiferal response to experimentally induced Erika oil pollution. Marine Micropaleontology.

2006;61: 76–93. doi:10.1016/j.marmicro.2006.05.005

10. Alve E, Korsun S, Schönfeld J, Dijkstra N, Golikova E, Hess S, et al. Foram-

AMBI: A sensitivity index based on benthic foraminiferal faunas from North-East Atlantic and Arctic fjords, continental shelves and slopes. Marine Micropaleontology.

2016;122: 1–12.

11. Schönfeld J, Alve E, Geslin E, Jorissen F, Korsun S, Spezzaferri S. The FOBIMO (FOraminiferal BIo-MOnitoring) initiative—Towards a standardised protocol for soft-bottom benthic foraminiferal monitoring studies. Marine Micropaleontology.

2012;94–95: 1–13. doi:10.1016/j.marmicro.2012.06.001

12. Jorissen F, Nardelli MP, Almogi-Labin A, Barras C, Bergamin L, Bicchi E, et al. Developing Foram-AMBI for biomonitoring in the Mediterranean: Species assignments to ecological categories. Marine Micropaleontology. 2018;140: 33–45.
13. Bouchet VM, Frontalini F, Francescangeli F, Sauriau P-G, Geslin E, Martins MVA, et al. Indicative value of benthic foraminifera for biomonitoring: Assignment to ecological groups of sensitivity to total organic carbon of species from European intertidal areas and transitional waters. Marine Pollution Bulletin. 2021;164: 112071.
14. Jorissen FJ, Fouet MPA, Singer D, Howa H. The Marine Influence Index

(MII): A Tool to Assess Estuarine Intertidal Mudflat Environments for the Purpose of

Foraminiferal Biomonitoring. Water. 2022;14: 676. doi:10.3390/w14040676

15. O’Brien PA, Polovodova Asteman I, Bouchet VM. Benthic foraminiferal indices and environmental quality assessment of transitional waters: a review of current challenges and future research perspectives. Water. 2021;13: 1898.
16. Elliott M, Quintino V. The Estuarine Quality Paradox, Environmental Homeostasis and the difficulty of detecting anthropogenic stress in naturally stressed areas. Marine Pollution Bulletin. 2007;54: 640–645. doi:10.1016/j.marpolbul.2007.02.003
17. Dauvin J-C, Vallet C, Mouny P, Zouhiri S. Main characteristics of the boundary layer macrofauna in the English Channel. In: Liebezeit G, Dittmann S, Kröncke I, editors. Life at Interfaces and Under Extreme Conditions. Dordrecht:

Springer Netherlands; 2000. pp. 139–156. doi:10.1007/978-94-011-4148-2_13

18. Hubas C, Davoult D, Cariou T, Artigas L. Factors controlling benthic metabolism during low tide along a granulometric gradient in an intertidal bay (Roscoff

Aber Bay, France). Mar Ecol Prog Ser. 2006;316: 53–68. doi:10.3354/meps316053

19. De Bettignies F, Dauby P, Lepoint G, Riera P, Bocher E, Bohner O, et al. Temporal succession of a macrofaunal community associated with kelp fragment accumulations in an in situ experiment. Mar Ecol Prog Ser. 2020;656: 109–121. doi:10.3354/meps13391
20. Chauris L. L’anse de l’Aber en Roscoff. Un ancien marais littoral envahi par la mer. Penn ar Bed (Brest). 1988;18: 1–11.
21. Hourdez S, Boidin-Wichlacz C, Jollivet D, Massol F, Rayol MC, Bruno R, et al. Investigation of Capitella spp. symbionts in the context of varying anthropic pressures: First occurrence of a transient advantageous epibiosis with the giant bacteria Thiomargarita sp. to survive seasonal increases of sulfides in sediments.

Science of the Total Environment. 2021;798: 149149.

22. Guilloux L, Rigaut-Jalabert F, Jouenne F, Ristori S, Viprey M, Not F, et al. An annotated checklist of Marine Phytoplankton taxa at the SOMLIT-Astan time series off Roscoff (Western English Channel, France): data collected from 2000 to 2010. Cah Biol Mar. 2013;54: 247–256.
23. Caracciolo M, Rigaut Jalabert F, Romac S, Mahé F, Forsans S, Gac J, et al.

Seasonal dynamics of marine protist communities in tidally mixed coastal waters.

Molecular Ecology. 2022;31: 3761–3783.

24. Rombouts I, Simon N, Aubert A, Cariou T, Feunteun E, Guérin L, et al. Changes in marine phytoplankton diversity: Assessment under the Marine Strategy Framework Directive. Ecological Indicators. 2019;102: 265–277.
25. Stagnol D, Renaud M, Davoult D. Effects of commercial harvesting of intertidal macroalgae on ecosystem biodiversity and functioning. Estuarine, Coastal and Shelf Science. 2013;130: 99–110.
26. Migné A, Bordeyne F, Davoult D. Long-term survey of intertidal rocky shore macrobenthic community metabolism and structure after primary succession. 2023. REFERENCE??
27. Riera P, Escaravage C, Leroux C. Trophic ecology of the rocky shore community associated with the Ascophyllum nodosum zone (Roscoff, France): a δ13C vs δ15N investigation. Estuarine, Coastal and Shelf Science. 2009;81: 143–148.
28. Boidin-Wichlacz C, Andersen AC, Jouy N, Hourdez S, Tasiemski A. A single coelomic cell type is involved in both immune and respiratory functions of the coastal bioindicator annelid: Capitella C-Channel1 from the English Channel. Developmental & Comparative Immunology. 2024;153: 105132.
29. Leclerc J, Riera P, Noël LM, Leroux C, Andersen AC. Trophic ecology of P omatoschistus microps within an intertidal bay (R oscoff, F rance), investigated through gut content and stable isotope analyses. Marine Ecology. 2014;35: 261–270.
30. Riera P. Trophic plasticity of the gastropod Hydrobia ulvae within an intertidal bay (Roscoff, France): A stable isotope evidence. Journal of Sea Research. 2010;63:

78–83.

31. Riera P, Hubas C. Trophic ecology of nematodes from various microhabitats of the Roscoff Aber Bay (France): importance of stranded macroalgae evidenced through δ13C and δ15N. Mar Ecol Prog Ser. 2003;260: 151–159. doi:10.3354/meps260151
32. Goldstein ST, Richardson EA. Fine structure of the foraminifer Haynesina germanica (Ehrenberg) and its sequestered chloroplasts. Marine Micropaleontology.

2018;138: 63–71. doi:10.1016/j.marmicro.2017.10.010

33. Christa G. Kleptoplasty. Current Biology. 2023;33: R465–R467. doi:10.1016/j.cub.2023.03.036
34. Rullier F. Etude bionomique de l’Aber de Roscoff. Université de Paris, Station biologique de Roscoff; 1959.
35. Heip C, Magda V, Guido V. The ecology of marine nematodes.

Oceanography and Marine Biology: an annual review. 1985;23: 399–489.

36. Liénart C, Savoye N, David V, Ramond P, Rodriguez Tress P, Hanquiez V, et al. Dynamics of particulate organic matter composition in coastal systems: Forcing of spatio-temporal variability at multi-systems scale. Progress in Oceanography.

2018;162: 271–289. doi:10.1016/j.pocean.2018.02.026

37. Dessandier P-A, Bonnin J, Kim J-H, Bichon S, Grémare A, Deflandre B, et al.

Lateral and vertical distributions of living benthic foraminifera off the Douro River

(western Iberian margin): Impact of the organic matter quality. Marine

Micropaleontology. 2015;120: 31–45. doi:10.1016/j.marmicro.2015.09.002

38. Zapata M, Rodríguez F, Garrido J. Separation of chlorophylls and carotenoids from marine phytoplankton:a new HPLC method using a reversed phase C8 column and pyridine-containing mobile phases. Mar Ecol Prog Ser. 2000;195: 29–45. doi:10.3354/meps195029
39. Coplen TB. Guidelines and recommended terms for expression of stableisotope-ratio and gas-ratio measurement results. Rapid Communications in Mass

Spectrometry. 2011;25: 2538–2560. doi:10.1002/rcm.5129

40. Shannon C, Weaver W. The Mathematical Theory of Communication.

Urbana: Illinois Press. 1964.

41. Buzas MA, Gibson TG. Species diversity: benthonic foraminifera in western North Atlantic. Science. 1969;163: 72–75.
42. Hammer Ø, Harper DAT, Ryan PD. PAST - PAlaeontological STatistics, ver.

1.89. Palaeontol electron. 2001;4: 1–9.

43. Schindelin J, Arganda-Carreras I, Frise E, Kaynig V, Longair M, Pietzsch T, et al. Fiji: an open-source platform for biological-image analysis. Nature methods. 2012;9:

676–682.

44. Legendre P, Legendre L. Numerical ecology. Elsevier; 2012.
45. Jorissen FJ, de Stigter HC, Widmark JGV. A conceptual model explaining benthic foraminiferal microhabitats. Marine Micropaleontology. 1995;26: 3–15. doi:10.1016/0377-8398(95)00047-X
46. Fontanier C, Jorissen FJ, Licari L, Alexandre A, Anschutz P, Carbonel P. Live benthic foraminiferal faunas from the Bay of Biscay: faunal density, composition, and microhabitats. Deep Sea Research Part I: Oceanographic Research Papers. 2002;49:

751–785. doi:10.1016/S0967-0637(01)00078-4

47. Armynot du Châtelet É, Bout-Roumazeilles V, Riboulleau A, Trentesaux A. Sediment (grain size and clay mineralogy) and organic matter quality control on living benthic foraminifera. Revue de Micropaléontologie. 2009;52: 75–84. doi:10.1016/j.revmic.2008.10.002
48. Mojtahid M, Geslin E, Coynel A, Gorse L, Vella C, Davranche A, et al. Spatial distribution of living (Rose Bengal stained) benthic foraminifera in the Loire estuary

(western France). Journal of Sea Research. 2016;118: 1–16. doi:10.1016/j.seares.2016.02.003

49. Fouet MPA, Singer D, Coynel A, Héliot S, Howa H, Lalande J, et al. Foraminiferal Distribution in Two Estuarine Intertidal Mudflats of the French Atlantic

Coast: Testing the Marine Influence Index. Water. 2022;14: 645.

doi:10.3390/w14040645

50. Ouisse V, Riera P, Migné A, Leroux C, Davoult D. Freshwater seepages and ephemeral macroalgae proliferation in an intertidal bay: I Effect on benthic community structure and food web. Estuarine, Coastal and Shelf Science. 2011;91: 272–281.

doi:10.1016/j.ecss.2010.10.034

51. Migné A, Ouisse V, Hubas C, Davoult D. Freshwater seepages and ephemeral macroalgae proliferation in an intertidal bay: II. Effect on benthic biomass and metabolism. Estuarine, Coastal and Shelf Science. 2011;92: 161–168. doi:10.1016/j.ecss.2010.12.023
52. Wilkinson M, Telfer T, Grundy S. Geographical variation in the distributions of macroalgae in estuaries. Netherlands Journal of Aquatic Ecology. 1995;29: 359–368. doi:10.1007/BF02084235
53. Alve E, Murray JW. Marginal marine environments of the Skagerrak and

Kattegat: a baseline study of living (stained) benthic foraminiferal ecology. Palaeogeography, Palaeoclimatology, Palaeoecology. 1999;146: 171–193.

doi:10.1016/S0031-0182(98)00131-X

54. Murray JW. Ecology and applications of benthic foraminifera. Cambridge university press; 2006.
55. Fontanier C, Mamo B, Dubosq N, Lamarque B, Rigaud S, Schmidt S, et al.

Seasonal variability of living benthic foraminifera from the West-Gironde mud patch

(Bay of Biscay, NE Atlantic): Three contrasted periods under the stereomicroscope.

Continental Shelf Research. 2023;268: 105117. doi:10.1016/j.csr.2023.105117

56. Kitazato H, Nakatsuka T, Shimanaga M, Kanda J, Soh W, Kato Y, et al. Longterm monitoring of the sedimentary processes in the central part of Sagami Bay, Japan: rationale, logistics and overview of results. Progress in Oceanography. 2003;57: 3–16. doi:10.1016/S0079-6611(03)00047-8
57. Nomaki H. Behavior and response of deep-sea benthic foraminifera to freshly supplied organic matter: a laboratory feeding experiment in microcosm environments. The Journal of Foraminiferal Research. 2005;35: 103–113. doi:10.2113/35.2.103
58. Mendes I, Rosa F, Dias J, Schönfeld J, Ferreira Ó, Pinheiro J. Inner shelf paleoenvironmental evolution as a function of land–ocean interactions in the vicinity of the Guadiana River, SW Iberia. Quaternary International. 2010;221: 58–67.
59. Armynot du Châtelet E, Debenay J-P, Soulard R. Foraminiferal proxies for pollution monitoring in moderately polluted harbors. Environmental Pollution. 2004;127:

27–40. doi:10.1016/S0269-7491(03)00256-2

60. Martins VA, Frontalini F, Tramonte KM, Figueira RCL, Miranda P, Sequeira C, et al. Assessment of the health quality of Ria de Aveiro (Portugal): Heavy metals and benthic foraminifera. Marine Pollution Bulletin. 2013;70: 18–33. doi:10.1016/j.marpolbul.2013.02.003
61. Alve E. Benthic foraminifera in sediment cores reflecting heavy metal pollution in Sorfjord, western Norway. The Journal of Foraminiferal Research. 1991;21: 1–19. doi:10.2113/gsjfr.21.1.1
62. Frontalini F, Buosi C, Da Pelo S, Coccioni R, Cherchi A, Bucci C. Benthic foraminifera as bio-indicators of trace element pollution in the heavily contaminated

Santa Gilla lagoon (Cagliari, Italy). Marine Pollution Bulletin. 2009;58: 858–877. doi:10.1016/j.marpolbul.2009.01.015

63. Dauvin J-C. Paradox of estuarine quality: Benthic indicators and indices, consensus or debate for the future. Marine Pollution Bulletin. 2007;55: 271–281.

doi:10.1016/j.marpolbul.2006.08.017

64. Atkinson K. The marine flora and fauna of the Isles of Scilly Foraminifera.

Journal of Natural History. 1970;4: 387–398. doi:10.1080/00222937000770371

65. Schönfeld J. A new benthic foraminiferal proxy for near-bottom current velocities in the Gulf of Cadiz, northeastern Atlantic Ocean. Deep Sea Research Part I:

Oceanographic Research Papers. 2002;49: 1853–1875.

66. Phipps M, Jorissen F, Pusceddu A, Bianchelli S, De Stigter H. Live benthic foraminiferal faunas along a bathymetrical transect (282-4987 M) on the portuguese margin (NE Atlantic). Journal of Foraminiferal Research. 2012;42: 66–81. doi:10.2113/gsjfr.42.1.66
67. Dessandier P, Bonnin J, Kim J, Bichon S, Deflandre B, Grémare A, et al. Impact of organic matter source and quality on living benthic foraminiferal distribution on a river dominated continental margin: A study of the Portuguese margin. JGR Biogeosciences. 2016;121: 1689–1714. doi:10.1002/2015JG003231

# Supporting information

**S 1 Fig 1. This is the S 1 Fig 1 SOMLIT data for salinity, oxygen, pH and Chl *a***. This is the S 1 Fig 1. SOMLIT data for salinity, oxygen, pH and Chl *a* over the sampling period from November 2021 to August 2022 in the ESTACADE Somlit sampling point. The data was extracted from the SOMLIT database (Service d'Observation en Milieu Littoral; www.somlit.fr) on 5 October 2022.

**S 2 Fig 2. This is the S 2 Fig 2 Rainfall data for Morlaix.** This is the **S 2 Fig 2**.

Rainfall data for Morlaix (21 km of Roscoff Aber Bay in Brittany, France) for the period October 2021 to August 2022 from the infoclimat.fr/climatologie/globale/31aout/morlaix/000AW.html website.

**S 3 Fig 3. This is the S 3 Fig 3 Plate of *Haneysina Germanica* foraminifera to compare brighfield and red fluorescence channel.** This is the S 3 Fig 3. Plate of *Haneysina Germanica* foraminifera at station 4 in November 2022 in brighfield (A) and red fluorescence channel (Ex 559-585nm, Em 600-690nm, BeamSplitter 590nm) (B) and Illustration of a density plot for red fluorescence (C).

**S 4 Fig 4. This is the S 4 Fig 4 Scanning electron micrographs of *Bolivina sp*.** This is the S 4 Fig 4. SEM image of *Bolivina sp.* in August 2022 at station 6 with a morphological anomaly of a double aperture.

figure 1	[Click here to access/download;Figure;Fig 1.tif](https://www.editorialmanager.com/pone/download.aspx?id=35525796&guid=30d6fdc8-c069-4412-baf1-da677da02bde&scheme=1)

Table 1	[Click here to access/download;Figure;tableau 1.ti](https://www.editorialmanager.com/pone/download.aspx?id=35525835&guid=dc6378ca-5047-440c-acf6-294b8d030937&scheme=1)

[Click here to access/download](https://www.editorialmanager.com/pone/download.aspx?id=35525828&guid=9f732fb5-90d2-4b41-a0b3-37c53e6333fe&scheme=1)

[Supporting Information](https://www.editorialmanager.com/pone/download.aspx?id=35525828&guid=9f732fb5-90d2-4b41-a0b3-37c53e6333fe&scheme=1)

[S 1 Fig 1.tif](https://www.editorialmanager.com/pone/download.aspx?id=35525828&guid=9f732fb5-90d2-4b41-a0b3-37c53e6333fe&scheme=1)

[Click here to access/download](https://www.editorialmanager.com/pone/download.aspx?id=35525829&guid=22103f6c-6af8-4c04-80c7-9a7e85d0586e&scheme=1)

[Supporting Information](https://www.editorialmanager.com/pone/download.aspx?id=35525829&guid=22103f6c-6af8-4c04-80c7-9a7e85d0586e&scheme=1)

[S 2 Fig 2.tif](https://www.editorialmanager.com/pone/download.aspx?id=35525829&guid=22103f6c-6af8-4c04-80c7-9a7e85d0586e&scheme=1)

[Click here to access/download](https://www.editorialmanager.com/pone/download.aspx?id=35525830&guid=9f052dde-4df6-43df-9318-cfe11c55dace&scheme=1)

[Supporting Information](https://www.editorialmanager.com/pone/download.aspx?id=35525830&guid=9f052dde-4df6-43df-9318-cfe11c55dace&scheme=1)

[S 3 Fig 3.tif](https://www.editorialmanager.com/pone/download.aspx?id=35525830&guid=9f052dde-4df6-43df-9318-cfe11c55dace&scheme=1)

[Click here to access/download](https://www.editorialmanager.com/pone/download.aspx?id=35525832&guid=d9aa87e2-4ca4-4c49-8c5a-226d8192f155&scheme=1)

[Supporting Information](https://www.editorialmanager.com/pone/download.aspx?id=35525832&guid=d9aa87e2-4ca4-4c49-8c5a-226d8192f155&scheme=1)

[S 4 Fig 4.tif](https://www.editorialmanager.com/pone/download.aspx?id=35525832&guid=d9aa87e2-4ca4-4c49-8c5a-226d8192f155&scheme=1)
